# Nitrification inhibitor chlorate and nitrogen substrates differentially affect comammox *Nitrospira* in a grassland soil

**DOI:** 10.3389/fmicb.2024.1392090

**Published:** 2024-05-14

**Authors:** Anish S. Shah, Pei-Chun Hsu, Chris Chisholm, Andriy Podolyan, Keith Cameron, Jiafa Luo, Roland Stenger, Sam Carrick, Wei Hu, Scott A. Ferguson, Wenhua Wei, Jupei Shen, Limei Zhang, Hongbin Liu, Tongke Zhao, Wenxue Wei, Weixin Ding, Hong Pan, Yimeng Liu, Bowen Li, Jianjun Du, Hong J. Di

**Affiliations:** ^1^Centre for Soil and Environmental Research, Lincoln University, Lincoln, New Zealand; ^2^AgResearch, Hamilton, New Zealand; ^3^Lincoln Agritech, Ruakura Research Centre, Hamilton, New Zealand; ^4^Manaaki Whenua - Landcare Research, Lincoln, New Zealand; ^5^The New Zealand Institute for Plant and Food Research, Lincoln, New Zealand; ^6^Department of Microbiology, University of Otago, Dunedin, New Zealand; ^7^Department of Biochemistry, University of Otago, Dunedin, New Zealand; ^8^Fujian Normal University, Fuzhou, China; ^9^Research Centre for Eco-Environmental Science, Chinese Academy of Sciences, Beijing, China; ^10^Institute of Agricultural Resources and Regional Planning, Chinese Academy of Agricultural Sciences, Beijing, China; ^11^Beijing Academy of Agriculture and Forestry Sciences, Beijing, China; ^12^Institute of Subtropical Agricultural Ecology, Chinese Academy of Sciences, Changsha, China; ^13^Institute of Soil Science, Chinese Academy of Sciences, Nanjing, China; ^14^College of Natural Resources and Environment, Shandong Agricultural University, Taian, China; ^15^Centre for Innovation and Development, Beijing Normal University, Zhuhai, China; ^16^College of Natural Resources and Environment, Hebei Agricultural University, Baoding, China; ^17^College of Resources and Environment, Zhongkai University of Agriculture and Engineering, Guangzhou, China

**Keywords:** comammox *Nitrospira*, ammonia oxidising archaea (AOA), ammonia oxidising bacteria (AOB), nitrification inhibitors, qPCR (quantitative PCR)

## Abstract

**Introduction:**

Through the combined use of two nitrification inhibitors, Dicyandiamide (DCD) and chlorate with nitrogen amendment, this study aimed to investigate the contribution of comammox *Nitrospira* clade B, ammonia oxidizing bacteria (AOB) and archaea (AOA) to nitrification in a high fertility grassland soil, in a 90-day incubation study.

**Methods:**

The soil was treated with nitrogen (N) at three levels: 0 mg-N kg^-1^ soil, 50 mg-N kg^-1^ soil, and 700 mg-N kg^-1^ soil, with or without the two nitrification inhibitors. The abundance of comammox *Nitrospira*, AOA, AOB, and nitrite oxidising bacteria (NOB) was measured using qPCR. The comammox Nitrospira community structure was assessed using Illumina sequencing.

**Results and Discussion:**

The results showed that the application of chlorate inhibited the oxidation of both NH_4_^+^ and NO_2_^-^ in all three nitrogen treatments. The application of chlorate significantly reduced the abundance of comammox *Nitrospira amo*A and *nxr*B genes across the 90-day experimental period. Chlorate also had a significant effect on the beta diversity (Bray-Curtis dissimilarity) of the comammox *Nitrospira* clade B community. Whilst AOB grew in response to the N substrate additions and were inhibited by both inhibitors, AOA showed litle or no response to either the N substrate or inhibitor treatments. In contrast, comammox *Nitrospira* clade B were inhibited by the high ammonium concentrations released from the urine substrates. These results demonstrate the differential and niche responses of the three ammonia oxidising communities to N substrate additions and nitrification inhibitor treatments. Further research is needed to investigate the specificity of the two inhibitors on the different ammonia oxidising communities.

## Graphical abstract

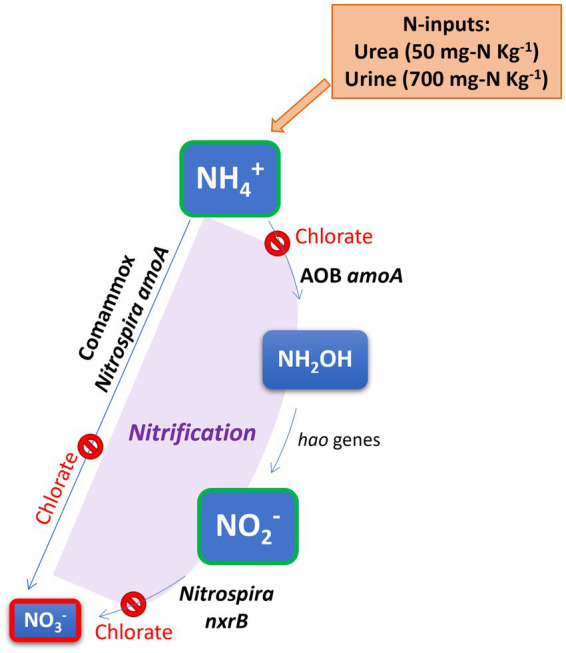

Graphical abstract showing the main components of the genes involved in the nitrification pathways. Chlorate application caused NH_4_^+^ and NO_2_^−^ to accumulate, which in turn caused NO_3_^−^ levels to reduce. Genes In bold Are those analysed in this study.

## 1 Introduction

Excessive fertiliser inputs along with the application of excess nitrogen (N) via the deposition of animal urine (Di and Cameron, 2000; Di et al., 2009) render grazed dairy pastures as the subject of scrutiny and concern due to nitrate (NO_3_^−^) leaching into waterways and the emission of nitrous oxide (N_2_O), a potent greenhouse gas and the leading cause of ozone depletion (Ravishankara et al., 2009). Additionally, these N-inputs, coupled with agricultural practices, are known to strongly affect the abundance and structure of soil microbial communities (Domeignoz-Horta et al., 2015; Hartmann et al., 2015).

Nitrification, the microbial oxidation of ammonia (NH_3_) via nitrite (NO_2_^−^) to nitrate (NO_3_^−^), is an integral part of the terrestrial N cycle, as it contributes to the production of two environmentally significant products, nitrous oxide (N_2_O), and (NO_3_^−^). Typically, ammonia oxidising archaea (AOA) are found in oligotrophic, acidic and N-depleted environments (Könneke et al., 2005), while ammonia oxidising bacteria (AOB) are found in eutrophic, N-rich environments (Costa et al., 2006; Di et al., 2009). Traditionally, nitrification was thought to be a two-step process which involved the oxidation of ammonia to nitrite (by AOA and/or AOB) and the oxidation of nitrite to nitrate by nitrite oxidising bacteria (NOB). This notion was challenged when the term comammox (complete ammonia oxidiser) was coined by Costa et al. (2006) based on the kinetic theory of optimum pathway length. Following the hypothetical possibility of such microbe(s) existing, comammox *Nitrospira* bacteria were discovered in two independent studies (Daims et al., 2015; van Kessel et al., 2015).

Comammox *Nitrospira* and canonical *Nitrospira* can be distinguished by the presence of the genes encoding the ammonia monooxygenase (AMO) enzyme. Previous results have suggested that comammox *Nitrospira* has a competitive advantage in low ammonium environments and are preferentially adapted to oligotrophic environments (Kits et al., 2019; Yang et al., 2020; Sakoula et al., 2021). Furthermore, comammox *Nitrospira* can be separated into clade A and clade B based on the phylogeny of the *amoA* gene, a subunit of AMO. Since their discovery, extensive research has been conducted on the ecology and abundance of comammox *Nitrospira* in terrestrial ecosystems worldwide (Shi et al., 2018; Zhao et al., 2019; Xu et al., 2020; Li et al., 2021; Sun et al., 2021; Wang et al., 2021; Hsu et al., 2022; Chisholm et al., 2023). Some studies suggest that comammox *Nitrospira* actively contributes to the nitrification process in a nutrient rich environment (Li et al., 2019; Xu et al., 2020). However, the comammox *Nitrospira* community in these ecosystems is dominated by clade A, with clade B typically being undetectable. To date, the comammox *Nitrospira* community found in New Zealand soils consists almost entirely of clade B (Hsu et al., 2022; Chisholm et al., 2023).

Chemical nitrification inhibitors (NIs) have been used as a mitigation technology to limit nitrate leaching and N_2_O emissions. They work by disrupting the oxidation of NH_3_ to NO_2_^−^, mainly through the deactivation of the microbial AMO enzyme (Subbarao et al., 2006; Shen et al., 2013). To date, several different NIs have been used to evaluate their ability to inhibit the AMO enzyme, such as 3,4-dimethylpyrazole phosphate (DMPP) (Zerulla et al., 2001; Di and Cameron, 2011), nitrapyrin (Abbasi and Adams, 1998), allylsulfide (Juliette et al., 1993), and dicyandiamide (DCD) (Amberger, 1989; Di and Cameron, 2002, 2003, 2004; Di et al., 2010). Chlorate, however, specifically inhibits the second step of nitrification, the oxidation of NO_2_^−^ to NO_3_^−^ (Hynes and Knowles, 1983). Although not currently used as an NI in agricultural settings due to its phytotoxic effects on plants (Hofstra, 1977; Borges et al., 2004), chlorate can be used *in vitro* to reveal the contribution of different nitrifying microorganisms to the overall soil nitrification process. The effect of chlorate and other NIs on nitrifying bacteria has been previously studied in liquid cultures (Shen et al., 2013; Sun et al., 2022) and soil (Li et al., 2020).

To investigate the contribution of DCD and chlorate to the nitrification process, a double inhibitor incubation experiment was devised. Two nitrogen application rates were used to determine the effect of different rates of nitrogen inputs on comammox *Nitrospira* growth. The rates applied were selected to represent common N inputs found in New Zealand pasture based-dairy systems. These were urea-N applied at 50 kg N ha^−1^ and urine-N, a concentrated localised application of N equivalent to 700 kg N ha^−1^ to simulate a dairy cow urine deposition (Cameron et al., 2013). Depending on the stocking rate, cow urine patches are typically the primary source of nitrate leaching in New Zealand pasture-based dairy farms (Cameron et al., 2013). We hypothesized that application of NIs to soil samples treated with different rates of nitrogen will (i) change the nitrogen dynamics of ammonium, nitrite, and nitrate in the treated soils throughout the experiment; (ii) alter the abundance of key microbial communities involved in nitrification in soil (AOA, AOB, and comammox *Nitrospira*); and (iii) change the community structure (abundance and composition) of comammox *Nitrospira*.

## 2 Materials and methods

### 2.1 Soil sampling

Soil samples were collected in Autumn 2021 from the Lincoln University Research Dairy Farm (LURDF) (43°38′26″ S, 172°26′37″ E) located on the South Island of New Zealand. This was the same study site used by Hsu et al. (2022). The area was excluded from grazing for at least 6 months before the sample collection. The soil type is Templeton silt loam [Typic Immature Pallic soil (Hewitt, 2010); USDA: Udic Haplustept, (Soil Survey, 2014)]. Soil samples (0–100 mm depth) were taken 5 m apart, randomly from six locations within the farm. Care was taken to avoid areas that were not representative, such as laneways, fence lines and animal campsites. After combining and mixing the six soil samples, organic debris, grass roots and loose gravel were removed, and the soil was sieved through a 5 mm sieve. A sub-sample weighing approximately 100 g was sent to Analytical Research Laboratories (ARL), Napier, New Zealand, to analyse the physicochemical properties (Supplementary data S1).

### 2.2 *In vitro* soil microcosm experiment

Soil microcosms were set up similarly to those described in Hsu et al. (2022). Briefly, 585 g of soil (500 g dry soil equivalent) was placed into polypropylene plastic containers (1 L volume). Following this, the microcosms were treated with either no nitrogen (control; N0), urea (50 mg N kg^−1^ of soil; Urea50), or synthetic urine (700 mg N kg^−1^ of soil; Urine700) (Clough et al., 1998), and either no nitrification inhibitor, DCD (10 mg kg^−1^ of dry soil), chlorate (500 mg kg^−1^ of dry soil, applied as NaClO_3_), or DCD + chlorate. All treatments were applied in solution. In total there were 12 treatments (Control, Control+DCD, Control+chlorate, Control+DCD + chlorate, Urea50, Urea50 + DCD, Urea50 + chlorate, Urea50 + DCD + chlorate, Urine700, Urine700 + DCD, Urine700 + chlorate, Urine700 + DCD + chlorate), and four biological replicates per treatment. The lid of each microcosm had two holes (10 mm in diameter) to allow for sufficient gas exchange. The containers were randomly placed in an incubator (Binder GmbH, Germany) at a constant temperature of 12°C for 90 days to simulate autumn/winter local soil temperatures. Soil samples were taken 1, 7, 14, 30, 60, and 90 days after treatment application. Gravimetric soil water content was maintained at 40% w/w throughout the experiment and measured at each sampling date. Concentrations of NH_4_^+^-N, NO_3_^−^-N and NO_2_^−^-N were determined using a Flow Injection Analyser (FIA) (FOSS FIA star 5,000 triple channel analyser) (Analytical Services Team, Lincoln University).

### 2.3 DNA extraction and gene quantification using qPCR

Genomic DNA was extracted from the soils at each sampling day (batch) using the NucleoSpin Soil DNA extraction kit (Macherey-Nagel GmbH & Co., Germany), per the manufacturer’s instructions, using 0.25 g of soil. Each batch had 48 samples (12 treatments with 4 replicates). DNA was eluted in 100 μL of elution buffer (from the NucleoSpin kit), diluted 20-fold using Ultrapure™ water (Invitrogen™, Thermo Fisher Scientific Inc., United States), and stored at −20°C. Details of PCR primers and amplification efficiencies observed for all genes are listed in Table 1 (thermal profiles and reaction details are in Supplementary data S2). Standards for each gene (plasmid DNA) used in the qPCR analysis were prepared as outlined previously (Hsu et al., 2022). Standard curves for each gene quantification were generated using a series of 1:10 dilutions of amplicon standards over a range of concentrations from 10^1^ to 10^7^ copies per microlitre. Each qPCR run included soil DNA samples (48 samples × 6 batches = 288), corresponding standards, and no-template control reactions. All qPCRs were conducted on the QuantStudio™ 5 system using a 384-well format (Applied Biosystems, Thermo Fisher Scientific Inc., USA). Melt curve analysis was performed at the end of each qPCR run to confirm the reaction specificity.

**Table 1:** Details of primers used for qPCR and the amplification efficiencies for each gene.

Target gene	Primer name	Sequence (5′ - 3′)	Final primer concentration (nM)	Amplification efficiency (*R*^2^ > 0.99)	References
Archaeal *amoA*	Arch_amoAF	STAATGGTCTGGCTTAGACG	500	97%	Francis et al. (2005)
	Arch_amoAR	GCGGCCATCCATCTGTATGT	500		
Bacterial *amoA*	amoA 1F_mod	GGGGHTTYTACTGGTGGT	320	106%	Hornek et al. (2006)
	amoAr_i	CCCCTCNGNAAANCCTTCTTC	320		
Comammox *Nitrospira amoA*	ComamoA_F	AGGNGAYTGGGAYTTCTGG	400	101%	Zhao et al. (2019)
	ComamoA_R	CGGACAWABRTGAABCCCAT	400		
*Nitrospira nxrB*	nxrB_169F	TACATGTGGTGGAACA	500	86%	Pester et al. (2014)
	nxrB_638R	CGGTTCTGGTCRATCA	500		

Thermal profiles are provided in Supplementary data S2.

### 2.4 Statistical analyses

A two-way analysis of variance (ANOVA) followed by a Fishers LSD test was used to determine if an inhibitor or nitrogen significantly affected the abundance of comammox, AOA, AOB *amoA,* or *nxrB* at each sampling date (Genstat 22^nd^ edition, VSN International 2022). A two-way ANOVA followed by a Fishers LSD was also used to determine if an inhibitor or nitrogen significantly affected ammonium, nitrite, or nitrate concentrations at each sampling date (Genstat 22^nd^ edition, VSN International 2022). Graphs were plotted using the ‘ggplot2’ package (Wickham, 2016) in R v4.2.2 (R Core Team, 2022) through the RStudio platform (v 2022.07.0 + 548).

### 2.5 Illumina sequencing

PCR amplicons for comammox *Nitrospira amoA* gene were prepared using soil genomic DNA from the Day 60 sampling point (thermal profiles and reaction details in Supplementary data S3). Primers for the PCR were: ComamoA_F 5’ AGGNGAYTGGGAYTTCTGG, ComamoA_R 5’ CGGACAWABRTGAABCCCAT (Zhao et al., 2019). The MiSeq overhang adapters were: forward adapter 5’ TCGTCGGCAGCGTCAGATGTGTATAAGAGACAG, and reverse adapter 5’ GTCTCGTGGGCTCGGAGATGTGTATAAGAGACAG. PCR products were purified using Agencourt AMPure XP magnetic beads (Beckman Coulter, Australia), and the final purified products were eluted in 20 μL of 10 mM Tris–HCl buffer (pH 8.5). PCR products were quantified using the Qubit™ dsDNA BR assay kit (Invitrogen™) on a Qubit 2.0 Fluorometer (Life Technologies, Invitrogen™). PCR amplicons [48 environmental, 2 no-template-controls, and 2 positive controls (cloned plasmids)] were then sent for subsequent library preparation, and Next Generation Sequencing (NGS) using the Illumina MiSeq 2 × 300 bp paired-end platform (Massey Genome Service, Massey University, New Zealand). Data was cleaned, and processed using the DADA2 (Callahan et al., 2016) pipeline via the ‘dada2’ package in R. Alpha (Shannon’s H`, species richness and Pielou’s evenness) and beta diversity indices, analyses and plotting were conducted using functions in the ‘vegan’ package (Oksanen et al., 2022) in R. PERMANOVA test (Anderson, 2001) was performed on the Bray–Curtis dissimilarity matrix for beta diversity analysis, using the *‘adonis2’* function in the vegan package. Principal coordinate analysis (PCoA) based on the Bray–Curtis dissimilarity matrix was used to perform unconstrained ordination.

### 2.6 Phylogenetic analyses

Nucleotide sequences of the *amoA* Amplicon Sequence Variants (ASVs) were imported into Geneious Prime® v2022.2.1 (Biomatters Ltd.), and the sequences were then translated into protein sequences. The protein and reference sequences were aligned using MUSCLE alignment v3.8.425 (Edgar, 2004). Subsequently, a phylogenetic tree was constructed on the alignment using the MrBayes v3.2.6 (Huelsenbeck and Ronquist, 2001) plugin within Geneious Prime®. The WAG substitution model (Whelan and Goldman, 2001) was used to build the tree, with 1 million iterations, gamma-distributed rates, a sub-sampling frequency of 10,000 and a final standard deviation of <0.01.

## 3 Results

### 3.1 Inorganic nitrogen concentration

#### 3.1.1 Ammonium-N

**Figure 1:**
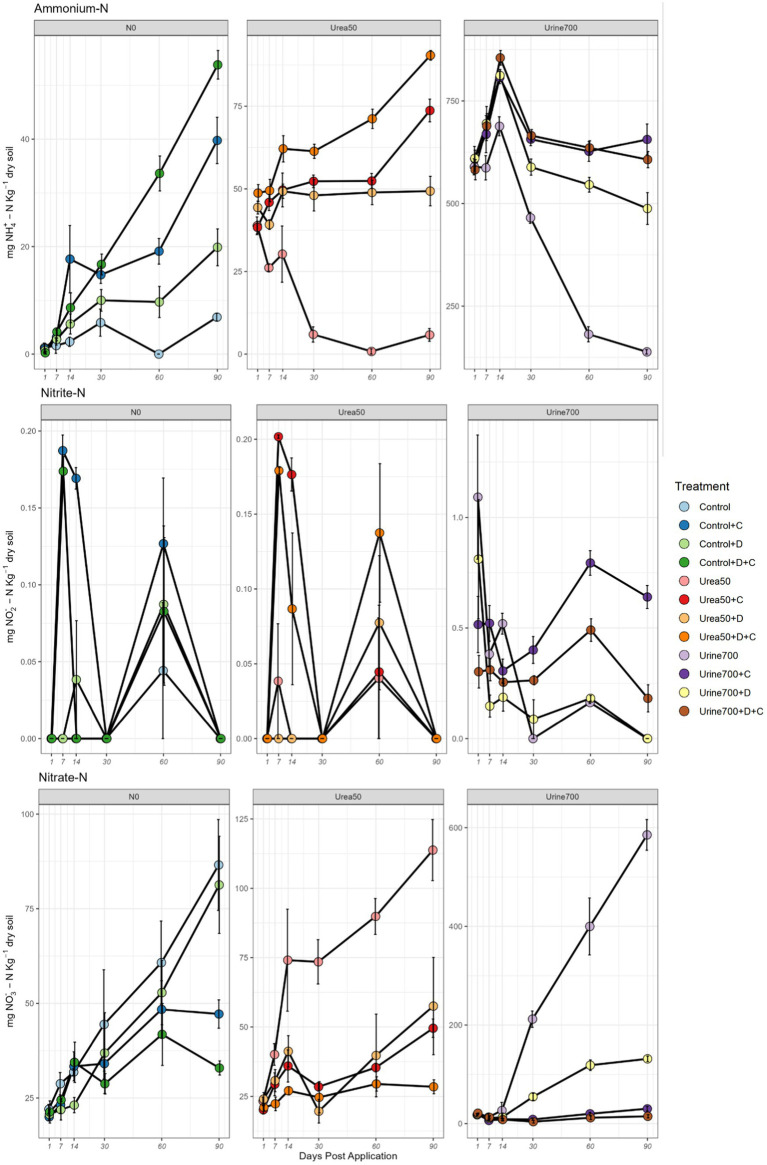
Nitrogen dynamics for NH_4_^+^-N, NO_2_^−^-N and NO_3_^−^-N for each of the treatments across the 90-day microcosm study. The three fertilization treatments (N0, Urea50 and Urine700) are shown on separate panels for each N-species. DCD, D; Chlorate, C. Vertical bars denote the standard error of the mean (*n* = 4).

Throughout the trial, soil ammonium concentrations were significantly higher in the Urine700 treatments than the Urea50 and Control treatments (*p* < 0.05). From day 14, both chlorate and DCD treated soils had higher soil ammonium concentrations than the respective treatments without DCD or chlorate. By day 60–90, almost all the urea- and urine- derived ammonium-N was depleted in soils without inhibitors present, compared to when chlorate and/or DCD were added. Soil ammonium concentrations were highest in the Urine700 + D + C treatment (855 mg NH_4_^+^-N kg dry soil) at day 14. Similarly, all other Urine700 treatments peaked at this day (Figure 1).

#### 3.1.2 Nitrite-N

The observed nitrite levels were at least two orders of magnitude lower than the ammonium and nitrate levels. Average soil nitrite concentrations ranged from 0 to 1.09 mg NO_2_^−^-N kg dry soil (peaking on day 1 for the Urine700 treatment). From day 30 onwards in the Urine700 fertilised soils, the chlorate treatment contained significantly more nitrite than the respective non-inhibitor treatments (*p* < 0.05). Throughout the trial, the concentration of nitrite in the DCD treatment were either similar or less than in the respective non-inhibitor treatments.

In the N0 and Urea50 treated soils, the nitrite was produced over the first 14 days then fully consumed by day 30, followed by another production burst, and full consumption by day 90. Throughout the trial, soils that received synthetic urine had a significantly higher concentration of nitrite than the Urea50 and N0 soils (*p* < 0.05) which did not significantly deviate from one another (Figure 1).

#### 3.1.3 Nitrate-N

A similar pattern of nitrate dynamics was observed across the three fertilizer treatments. Prior to day 14, the application of an inhibitor had no significant effect on the soil nitrate concentration (*p* > 0.05). In the non-fertilised soils (N0), from day 14, the Control and Control+DCD treatments showed a similar pattern, and by day 90 they contained significantly more nitrate than DCD and DCD + chlorate treated soils. From day 14 onwards, the non-inhibitor treatments in the Urea50 and Urine700 fertilised soils contained significantly more nitrate than soils treated with either or both DCD and chlorate. Furthermore, from day 30 in the Urine700 fertilised soils, the chlorate treatments (C and D + C) contained significantly less nitrate than the non-inhibitor and DCD-only treatments.

Throughout the trial, the application of nitrogen had a significant effect on the concentration of nitrate within the soil. However, from days 1–14 the Urea50 treated soils had a significantly higher concentration of nitrate than the Urine700 soils (*p* ≤ 0.05). From day 30, Urine700 contained significantly more nitrate than the Urea50 and Control treatments (Figure 1).

### 3.2 Gene abundance

#### 3.2.1 Comammox *Nitrospira amoA*

After day 14, comammox *Nitrospira amoA* gene abundance was highest in either the control or the Urea50 treatments, except for day 60, where it was highest in the Urine700 treatment. Commamox *Nitrospira* abundance in the Urine700 treatment was significantly lower than the control and Urea50 treatments at days 14, 30, and 90.

**Figure 2:**
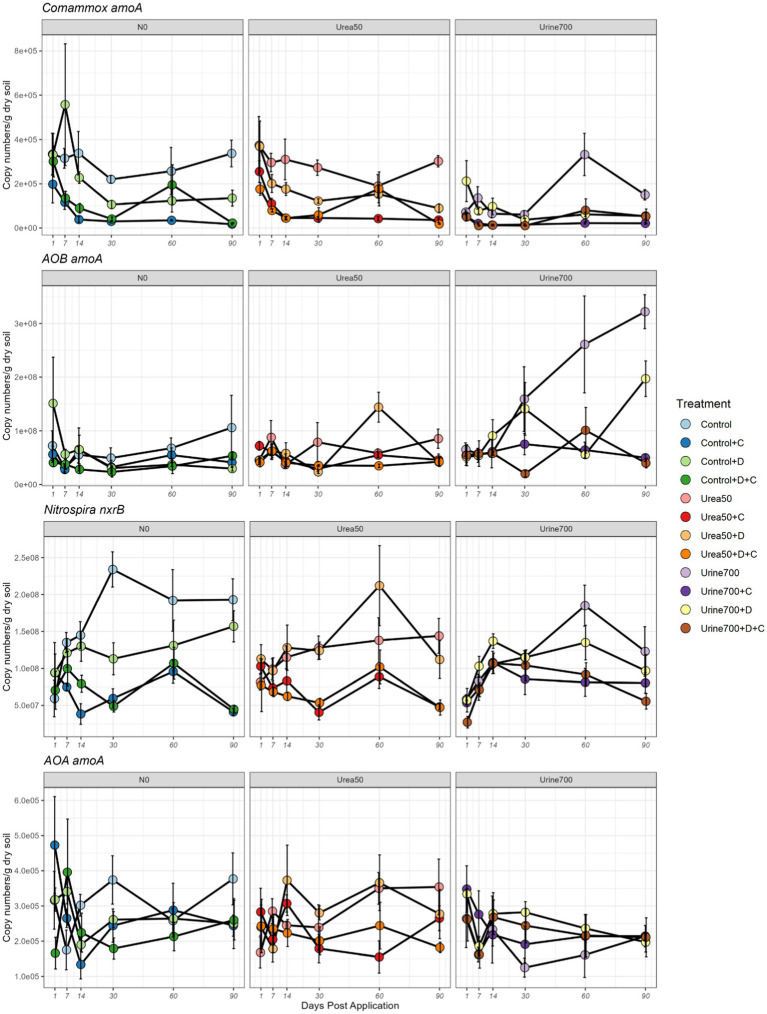
Microbial population dynamics for the gene abundance (copy numbers/g of dry soil) of comammox *Nitrospira amoA*, AOB *amoA, Nitrospira nxrB*, and AOA *amoA* (nitrification genes) across the 90-day microcosm experiment. The three fertilization treatments (N0, Urea50 and Urine700) are shown on separate panels for each N-species. DCD, D; Chlorate, C. Vertical bars denote the standard error of the mean (*n* = 4).

From day 7, comammox *Nitrospira amoA* gene abundance was significantly lower in the chlorate treatments than both the respective non-chlorate and DCD treatments (*p* < 0.05). Comammox *Nitrospira* abundance was also lower in the DCD treatment when compared to the respective non-DCD treatments (*p* < 0.05) (Figure 2).

#### 3.2.2 AOB *amoA*

AOB *amoA* gene abundance was highest at day 90 in the Urine700 treatment. From days 1–14, there was no significant difference in AOB *amoA* gene abundance. From day 30 onwards, AOB *amoA* gene abundance was highest in the Urine700 treatments. This was calculated to be significant (except for Urine700 + D at day 30) (*p* < 0.05).

After day 14, AOB *amoA* gene abundance was significantly lower in the chlorate and the DCD + chlorate treatments. AOB *amoA* gene abundance in the DCD treatment was only significantly lower than the respective non-DCD treatments at day 90 (Figure 2).

#### 3.2.3 Nitrospira *nxrB*

From day 14, the application of chlorate reduced *nxrB* gene abundance to below the DCD and control levels. At day 30, DCD was also shown to lower *nxrB* gene abundance when compared to the control. However, this difference was non-significant for the remainder of the trial. *nxrB* gene abundance was highest in the control treatment at day 30 (2.34 × 10^8^), followed by the Urea50 + D treatment and Urine700 treatment at day 60 (2.12 × 10^8^ and 1.85 × 10^8^, respectively). Overall, the application of nitrogen had no significant effect on *nxrB* gene abundance (Figure 2).

#### 3.2.4 AOA *amoA*

Throughout the trial, the application of nitrogen or inhibitor had little effect on AOA *amoA* gene abundance. However, the Urine700 treatment reduced AOA abundance from days 14–30 (Figure 2).

#### 3.2.5 Gene abundance and ammonium concentration

Since we found that the Urine700 treatment and application of NIs caused ammonium concentrations to increase (Figure 1), we further analyzed the relationships between the concentration of NH_4_^+^-N and the gene copy numbers for the nitrification genes (comammox *amoA*, *Nitrospira nxrB*, AOB *amoA* and AOA *amoA*) for each of the treatments. This was done to decipher which of the nitrifiers were active in the consumption of ammonium. The correlation test was conducted separately for the three nitrogen fertilization rates (Table 2; Supplementary Figures S1–S3). The application of chlorate caused a strong negative correlation between comammox *Nitrospira amoA* and NH_4_^+^-N in the N0 and Urea50 treatments. Interestingly, the abundance of AOB *amoA* showed a strong negative correlation with NH_4_^+^-N in the Urine700 treatment, without NI application. The observed negative correlation implies the consumption of the ammonium by the growing AOB populations. The application of chlorate and DCD + chlorate caused a strong positive correlation between *Nitrospira nxrB* and NH_4_^+^-N in the Urine700 treatment. These positive correlations were a result of decreasing NH_4_^+^-N levels and corresponding reduction in *Nitrospira nxrB* gene abundance. The abundance of AOA *amoA* gene showed no significant correlation with NH_4_^+^-N for any of the treatments.

**Table 2:** Spearman’s correlation (*ρ*) and corresponding *p* values for the relationship between nitrification gene copy numbers and ammonium concentration.

	Gene abundance vs. ammonium concentration			
N0	Comammox *amoA*	AOB *amoA*	*Nitrospira nxrB*	AOA *amoA*
Control	*ρ* = 0.048, *p* = 0.822	*ρ* = −0.196, *p* = 0.371	*ρ* = 0.295, *p* = 0.162	*ρ* = 0.345, *p* = 0.099
Control+D	*ρ* = −0.493, *p* = 0.014	*ρ* = −0.424, *p* = 0.039	*ρ* = 0.209, *p* = 0.328	*ρ* = −0.349, *p* = 0.094
Control+C	*ρ* = −0.730, *p* < 0.001	*ρ* = −0.006, *p* = 0.977	*ρ* = −0.185, *p* = 0.386	*ρ* = −0.173, *p* = 0.420
Control+D + C	*ρ* = −0.614, *p* = 0.001	*ρ* = 0.094, *p* = 0.662	*ρ* = −0.207, *p* = 0.331	*ρ* = 0.035, *p* = 0.870
Urea50				
Urea50	*ρ* = 0.251, *p* = 0.238	*ρ* = −0.251, *p* = 0.237	*ρ* = −0.347, *p* = 0.096	*ρ* = −0.272, *p* = 0.198
Urea50 + D	*ρ* = −0.117, *p* = 0.586	*ρ* = 0.106, *p* = 0.622	*ρ* = 0.004, *p* = 0.985	*ρ* = 0.307, *p* = 0.144
Urea50 + C	*ρ* = −0.653, *p* < 0.001	*ρ* = −0.376, *p* = 0.071	*ρ* = −0.619, *p* = 0.002	*ρ* = −0.156, *p* = 0.466
Urea50 + D + C	*ρ* = −0.528, *p* = 0.008	*ρ* = −0.308, *p* = 0.143	*ρ* = 0.078, *p* = 0.715	*ρ* = 0.049, *p* = 0.821
Urine700				
Urine700	*ρ* = −0.398, *p* = 0.054	*ρ* = −0.729, *p* < 0.001	*ρ* = −0.339, *p* = 0.105	*ρ* = 0.080, *p* = 0.709
Urine700 + D	*ρ* = 0.206, *p* = 0.334	*ρ* = −0.138, *p* = 0.518	*ρ* = 0.157, *p* = 0.464	*ρ* = 0.164, *p* = 0.443
Urine700 + C	*ρ* = −0.173, *p* = 0.420	*ρ* = 0.056, *p* = 0.793	*ρ* = 0.579, *p* = 0.003	*ρ* = −0.100, *p* = 0.640
Urine700 + D + C	*ρ* = −0.453, *p* = 0.026	*ρ* = 0.139, *p* = 0.525	*ρ* = 0.519, *p* = 0.009	*ρ* = 0.211, *p* = 0.320

Results are split for each of the three nitrogen treatments (no nitrogen = N0), averaged across the 90-day experiment. Values in red are significant negative correlations, whereas those in blue are significant positive correlations, and values in black are non-significant correlations.

### 3.3 Comammox *Nitrospira* amoA sequencing results

**Figure 3:**
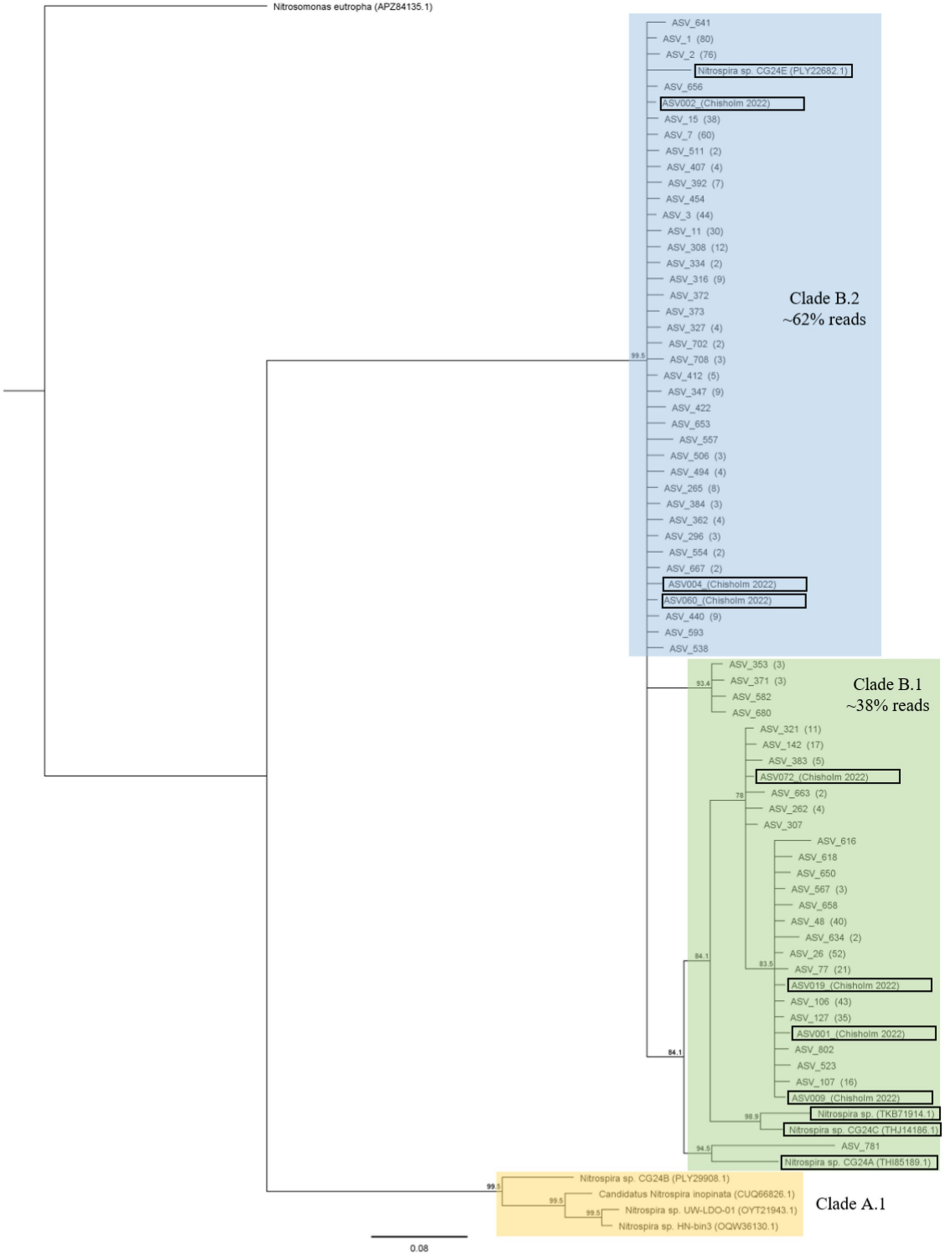
The consensus phylogenetic tree (≥70% support threshold) from 1502 raw trees constructed using the WAG model in MrBayes. Sixty-one unique representative protein sequences were used in the model (numbers in parentheses refer to the number of sequences which matched the representative sequence). Sequences are clustered into Clade B.1, Clade B.2, and reference Nitrospira sequences (belonging to Clade A.1). The tree also includes reference sequences from (Chisholm et al., 2023; Palomo et al., 2022; Zhao et al., 2021) (in black boxes), and is to scale with pairwise distances indicated on the branches. Nitrosomonas eutropha (accession number APZ84135.1) was used as the outgroup to root the tree.

Across the 48 environmental samples and the controls, 992 ASVs were obtained with 604,478 reads. Upon removing the controls and filtering out ASVs with <10 reads, 702 ASVs remained with 500,370 reads. The representative nucleotide sequences were translated into protein sequences for further analysis. From these 702 protein sequences, 61 unique sequences (141 amino acids in length) were obtained. These 61 sequences can be found under GenBank accession numbers OQ604566 to OQ604626. The sequences clustered into clade B.1 (267 sequences making up ~38% of total reads), and clade B.2 (435 sequences making up ~62% of total reads), as seen in the phylogenetic tree in Figure 3. No sequences belonging to clade A were found. There was no significant difference in alpha diversity (Shannon’s H`, species richness and Pielou’s evenness) across samples (Supplementary Figure S4). PERMANOVA analysis (999 permutations) showed that there was a significant effect (*p* = 0.015) of chlorate application on the Bray–Curtis dissimilarity (beta diversity) between comammox *Nitrospira amoA* communities (Supplementary Figure S5).

## 4 Discussion

This study focussed on how two nitrification inhibitors; DCD and chlorate, with and without the addition of nitrogen, affected AOA, AOB and comammox *Nitrospira* abundance and community composition. A comprehensive soil microcosm study across 90 days (six time points), with four replicates across 12 treatments was conducted. Shortly after the treatment application, chlorate was shown to significantly deter the growth of comammox *Nitrospira*. It is theorised that chlorate acts as a nitrite oxidiser inhibitor (Tatari et al., 2017). However, in this experiment, chlorate was also shown to inhibit AOB. Furthermore, high-throughput sequencing (using Illumina Miseq) revealed that only comammox *Nitrospira amoA* clade B sequences were present in the soil and that chlorate application affected the beta diversity of comammox *Nitrospira* clade B.

Traditionally, chlorate has been used to specifically inhibit the oxidation of nitrite to nitrate (Belser and Mays, 1980; Bauhus et al., 1996; Xu et al., 2011; Sun et al., 2022). This inhibitory effect is caused by reducing chlorate to chlorite, which inhibits NOB, thereby wholly blocking the oxidation of nitrite to nitrate, but does not affect ammonia oxidation to nitrite (Xu et al., 2011). Throughout this study, the application of chlorate significantly reduced comammox *Nitrospira amoA* and *Nitrospira nxrB* gene copy numbers, irrespective of ammonium concentration. Several studies have indicated that comammox *Nitrospira* may prefer a low nitrogen environment (Kits et al., 2017; Wang et al., 2019; Xu et al., 2020; He et al., 2021), partly due to MEP-type ammonia transporters (Palomo et al., 2018) and an extra non-operon *amoC* gene in their genome which are responsible for adaptation to low ammonia conditions (Palomo et al., 2018; Koch et al., 2019). An enrichment culture of comammox *Nitrospira* (clade A) was reported to exhibit a very high affinity towards ammonium and was inhibited by high ammonium levels (Sakoula et al., 2021). Although we did not find any clade A comammox *Nitrospira* sequences, our results also suggest that comammox *Nitrospira* clade B may be inhibited by high ammonium levels, since its abundance had a strong negative correlation with ammonium concentration, particularly when chlorate was applied (Table 2). In contrast, canonical *Nitrospira* were shown to be inhibited by relatively low concentrations of free ammonia (Anthonisen et al., 1976; Park and Bae, 2009). Therefore, it is possible that ammonia has a similar effect on comammox *Nitrospira*, thereby implying that comammox *Nitrospira* clade B may also behave like canonical *Nitrospira*.

From days 1–60, the abundance of comammox *Nitrospira* in the Urine700 treatment was significantly lower than the control. This may have been due to the accumulation of ammonia, or the rapid increase in soil pH due to the hydrolysis of urea (Curtin et al., 2020), both of which have been shown to be negatively associated with comammox *Nitrospira* abundance (Shi et al., 2018; Sun et al., 2021; Hsu et al., 2022). However, at day 60, comammox *Nitrospira* abundance increased in the Urine700 treatment. This may be because of the significant decrease in soil pH and reduction in soil ammonium concentrations associated with nitrification, both of which would reduce available ammonia to levels that are theoretically more suited to comammox *Nitrospira* (Kits et al., 2017; Palomo et al., 2018; Koch et al., 2019). This suggests that comammox *Nitrospira* may play a small role in the production of nitrate from urine deposition. Further research is needed to confirm or reject this hypothesis.

From day 30, AOB *amoA* gene abundance was significantly higher in the Urine700 treatments than the control. It has been well-established in previous studies that AOB respond positively to the application of urine/high concentration of nitrogen (Nicol et al., 2008; Di et al., 2009, 2010; Di and Cameron, 2016; Ouyang et al., 2016). This was because of the drastic and significant increase in soil ammonia/ammonium concentration, which is linked to AOB abundance (Ouyang et al., 2016). Interestingly, the application of DCD did not significantly affect AOB abundance until day 90. This is in contrast to previous results, which have shown that DCD significantly inhibits the growth of AOB (Di et al., 2014). Sun et al. (2022) reported that chlorate did not inhibit AOA and AOB, but we found that chlorate significantly inhibited AOB abundance from day 30, which is beyond the duration of their study (conducted across 28 days). This was also in contrast to previous results as chlorate was thought to be a specific nitrite oxidation inhibitor (Belser and Mays, 1980). However, some studies have questioned the selectivity of chlorate as a specific nitrification inhibitor (Hynes and Knowles, 1983; Tatari et al., 2017). Tatari et al. (2017) suggest that the selectivity of chlorate may be compromised by the type of NOB present and may be abolished when comammox *Nitrospira* dominate ammonia oxidation in the system. It is thought that the extracellular ClO_2_^−^ produced by the NOB reduction of chlorate can inhibit AOB (Hynes and Knowles, 1983). This explains why AOB began to be significantly inhibited by chlorate 23 days after comammox and canonical *Nitrospira.* The relatively quicker effect chlorate had on AOB abundance may be attributed to the mechanism of its inhibition. DCD is thought to bind to the ammonia monooxygenase active site, while chlorite (produced by the oxidation of chlorate by *Nitrospira*) is thought to inactivate the bacterium itself (Rungvetvuthivitaya et al., 2014). Therefore, it is possible that some metabolically diverse members of the AOB community may have persisted under the DCD treatment, thereby reducing its effect on overall community abundance.

Throughout the experiment, AOA was shown to be unaffected by nitrogen, DCD, and chlorate. This may be because of the difference in how their ammonia monooxygenase function, metabolic diversity, or inactivity in a high nutrient environment (Di et al., 2009; Hatzenpichler, 2012). In contrast, He et al. (2021) found that AOA growth ceased upon adding ammonium (i.e., high nitrogen environment). It is worthwhile to note that the primers He et al. (2021) used to detect AOA *amoA* were different to those used in this study, which may be a reason for the contrasting results.

Similar to Hsu et al. (2022), DCD was shown to inhibit comammox *Nitrospira* (only after day 60), although its effect was not as strong as chlorate. It is unclear whether the inhibition observed in the DCD treatments was caused by inactivation of the ammonia monooxygenase enzyme, or indirect inhibition caused by the accumulation of free ammonia. When DCD and chlorate were applied in combination, the reduction in gene abundance of comammox *Nitrospira amoA* and *Nitrospira nxrB* was driven by chlorate alone. Notably, for comammox *Nitrospira*, chlorate lowered the abundance by 4 to 5.5-fold, whereas DCD only reduced abundance by about 1.5-fold over controls (no NI added) across the three nitrogen treatments. Fu et al. (2018) found that the application of both DCD and chlorate significantly decreased nitrite oxidation, and that chlorate had a more substantial effect than DCD, which supports our findings; however, they only tested the impact of NIs over 7 days.

Illumina sequencing of the comammox *Nitrospira amoA* gene was conducted only on the day-60 samples, based on the qPCR results. Phylogenetic analysis of the comammox *Nitrospira amoA* gene revealed that only sequences belonging to clade B were found (Figure 3), which is consistent with previous studies on grazed pasture soils (Hsu et al., 2022; Chisholm et al., 2023). Beta diversity of clade B sequences was only affected by the application of chlorate (not DCD or the N-inputs), whereas alpha diversity was unaffected by any of the treatments.

Comammox *Nitrospira* harbour the genes that encode ammonia monooxygenase (*amoA*), hydroxylamine dehydrogenase (*hao*), and nitrite oxidoreductase (*nxr*) (Daims et al., 2015; van Kessel et al., 2015; Palomo et al., 2018). Based on the findings of this research, we postulate that comammox *Nitrospira* may be more active in the second step of the ammonia oxidation pathway, which involves nitrite oxidoreductase, notably because the application of chlorate alone (not DCD) reduced the abundance of the comammox *Nitrospira amoA* gene. Yet, nitrification inhibition by chlorate is complex, and care is needed to interpret the pathways inhibited by applying chlorate (Fu et al., 2018). The results of this study raise some concerns regarding the use of chlorate as a specific nitrite oxidation inhibitor.

## 5 Conclusion

The results supported our first hypothesis, in that the application of chlorate significantly increased the amount of ammonium and nitrite, and significantly decreased the amount of nitrate in the soils compared to DCD application or no added inhibitors. Our second and third hypotheses were also supported, in that chlorate was shown to significantly inhibit comammox *Nitrospira* and change its community structure (beta diversity). DCD was also shown to inhibit comammox *Nitrospira*, although its effect was not as strong as chlorate. It is unclear whether the inhibition observed in the DCD treatments was caused by inactivation of the ammonia monooxygenase enzyme, or indirect inhibition caused by the accumulation of free ammonia. After the ammonium concentration decreased in the Urine700 treatment, comammox *Nitrospira* abundance increased. This suggests that comammox *Nitrospira* growth might also have been restricted by the high ammonium concentrations following urine application. Interestingly, AOB abundance was also significantly inhibited by chlorate. This may have been due to the accumulation of extracellular ClO_2_^−^, which is thought to inactivate AOB. These results raise some concerns regarding the use of chlorate as a specific nitrite oxidation inhibitor.

## Data availability statement

The data presented in this study is available in the GenBank repository, under accession numbers OQ604566 - OQ604626, which can be found in the PopSet 2633561121 (https://www.ncbi.nlm.nih.gov/popset/2633561121?report=genbank).

## Author contributions

AS: Writing – original draft, Visualization, Validation, Software, Methodology, Investigation, Formal analysis, Data curation. P-CH: Writing – review & editing, Methodology, Investigation, Formal analysis, Data curation. CC: Validation, Software, Writing – review & editing, Formal analysis. AP: Writing – review & editing, Supervision, Project administration. KC: Writing – review & editing, Supervision, Resources, Project administration, Funding acquisition, Conceptualization. JL: Writing – review & editing, Resources, Project administration, Funding acquisition. RS: Writing – review & editing, Resources, Funding acquisition. SC: Writing – review & editing, Funding acquisition. WH: Writing – review & editing, Funding acquisition. SF: Writing – review & editing, Funding acquisition. WHW: Writing – review & editing, Funding acquisition. JS: Writing – review & editing, Methodology, Funding acquisition. LZ: Writing – review & editing, Methodology, Funding acquisition. HL: Writing – review & editing, Funding acquisition. TZ: Writing – review & editing, Funding acquisition. WXW: Writing – review & editing, Funding acquisition. WD: Writing – review & editing, Funding acquisition. HP: Writing – review & editing, Funding acquisition. YL: Writing – review & editing, Funding acquisition. BL: Writing – review & editing, Funding acquisition. JD: Writing – review & editing, Funding acquisition. HD: Writing – review & editing, Resources, Project administration, Methodology, Funding acquisition, Conceptualization.

## References

[ref1] Abbasi M. Adams W. (1998). Loss of nitrogen in compacted grassland soil by simultaneous nitrification and denitrification. Plant Soil 200, 265–277. doi: 10.1023/A:1004398520150

[ref2] Amberger A. (1989). Research on dicyandiamide as a nitrification inhibitor and future outlook. Commun. Soil Sci. Plant Anal. 20, 1933–1955. doi: 10.1080/00103628909368195

[ref3] Anderson M. J. (2001). A new method for non-parametric multivariate analysis of variance. Aust. Ecol. 26, 32–46. doi: 10.1111/j.1442-9993.2001.01070.pp.x

[ref4] Anthonisen A. Loehr R. Prakasam T. Srinath E. (1976). Inhibition of nitrification by ammonia and nitrous acid. Water Pollut. Control Federat. 48, 835–852. Available at: https://www.jstor.org/stable/25038971 948105

[ref5] Bauhus J. Meyer A. Brumme R. (1996). Effect of the inhibitors nitrapyrin and sodium chlorate on nitrification and N_2_O formation in an acid forest soil. Biol. Fertil. Soils 22, 318–325. doi: 10.1007/BF00334576

[ref6] Belser L. Mays E. (1980). Specific inhibition of nitrite oxidation by chlorate and its use in assessing nitrification in soils and sediments. Appl. Environ. Microbiol. 39, 505–510. doi: 10.1128/aem.39.3.505-510.1980, PMID: 16345525 PMC291368

[ref7] Borges R. Miguel E. C. Dias J. M. R. da Cunha M. Bressan-Smith R. E. de Oliveira J. G.. (2004). Ultrastructural, physiological and biochemical analyses of chlorate toxicity on rice seedlings. Plant Sci. 166, 1057–1062. doi: 10.1016/j.plantsci.2003.12.023

[ref8] Callahan B. J. McMurdie P. J. Rosen M. J. Han A. W. Johnson A. J. A. Holmes S. P. (2016). DADA2: high-resolution sample inference from Illumina amplicon data. Nat. Methods 13, 581–583. doi: 10.1038/nmeth.3869, PMID: 27214047 PMC4927377

[ref9] Cameron K. C. Di H. J. Moir J. L. (2013). Nitrogen losses from the soil/plant system: a review. Ann. Appl. Biol. 162, 145–173. doi: 10.1111/aab.12014

[ref10] Chisholm C. Di H. J. Cameron K. Podolyan A. Shah A. Hsu L.. (2023). Soil moisture is a primary driver of comammox *Nitrospira* abundance in New Zealand soils. Sci. Total Environ. 858:159961. doi: 10.1016/j.scitotenv.2022.159961, PMID: 36343813

[ref11] Clough T. J. Ledgard S. F. Sprosen M. S. Kear M. J. (1998). Fate of ^15^N labelled urine on four soil types. Plant Soil 199, 195–203. doi: 10.1023/A:1004361009708

[ref12] Costa E. Pérez J. Kreft J.-U. (2006). Why is metabolic labour divided in nitrification? Trends Microbiol. 14, 213–219. doi: 10.1016/j.tim.2006.03.006, PMID: 16621570

[ref13] Curtin D. Peterson M. Qiu W. Fraser P. (2020). Predicting soil pH changes in response to application of urea and sheep urine. J. Environ. Qual. 49, 1445–1452. doi: 10.1002/jeq2.20130, PMID: 33016443

[ref14] Daims H. Lebedeva E. V. Pjevac P. Han P. Herbold C. Albertsen M.. (2015). Complete nitrification by *Nitrospira* bacteria. Nature 528, 504–509. doi: 10.1038/nature16461, PMID: 26610024 PMC5152751

[ref15] Di H. J. Cameron K. C. (2000). Calculating nitrogen leaching losses and critical nitrogen application rates in dairy pasture systems using a semi-empirical model. N. Z. J. Agric. Res. 43, 139–147. doi: 10.1080/00288233.2000.9513415

[ref16] Di H. J. Cameron K. C. (2002). The use of a nitrification inhibitor, dicyandiamide (DCD), to decrease nitrate leaching and nitrous oxide emissions in a simulated grazed and irrigated grassland. Soil Use Manag. 18, 395–403. doi: 10.1111/j.1475-2743.2002.tb00258.x

[ref17] Di H. J. Cameron K. C. (2003). Mitigation of nitrous oxide emissions in spray-irrigated grazed grassland by treating the soil with dicyandiamide, a nitrification inhibitor. Soil Use Manag. 19, 284–290. doi: 10.1111/j.1475-2743.2003.tb00317.x

[ref18] Di H. J. Cameron K. C. (2004). Effects of temperature and application rate of a nitrification inhibitor, dicyandiamide (DCD), on nitrification rate and microbial biomass in a grazed pasture soil. Aust. J. Soil Res. 42, 927–932. doi: 10.1071/SR04050

[ref19] Di H. J. Cameron K. C. (2011). Inhibition of ammonium oxidation by a liquid formulation of 3, 4-Dimethylpyrazole phosphate (DMPP) compared with a dicyandiamide (DCD) solution in six new Zealand grazed grassland soils. J. Soils Sediments 11, 1032–1039. doi: 10.1007/s11368-011-0372-1

[ref20] Di H. J. Cameron K. C. (2016). Inhibition of nitrification to mitigate nitrate leaching and nitrous oxide emissions in grazed grassland: a review. J. Soils Sediments 16, 1401–1420. doi: 10.1007/s11368-016-1403-8

[ref21] Di H. J. Cameron K. C. Podolyan A. Robinson A. (2014). Effect of soil moisture status and a nitrification inhibitor, dicyandiamide, on ammonia oxidizer and denitrifier growth and nitrous oxide emissions in a grassland soil. Soil Biol. Biochem. 73, 59–68. doi: 10.1016/j.soilbio.2014.02.011

[ref22] Di H. J. Cameron K. C. Shen J.-P. Winefield C. S. O’Callaghan M. Bowatte S.. (2009). Nitrification driven by bacteria and not archaea in nitrogen-rich grassland soils. Nat. Geosci. 2, 621–624. doi: 10.1038/ngeo613

[ref23] Di H. J. Cameron K. C. Sherlock R. R. Shen J.-P. He J.-Z. Winefield C. S. (2010). Nitrous oxide emissions from grazed grassland as affected by a nitrification inhibitor, dicyandiamide, and relationships with ammonia-oxidizing bacteria and archaea. J. Soils Sediments 10, 943–954. doi: 10.1007/s11368-009-0174-x

[ref24] Domeignoz-Horta L. A. Spor A. Bru D. Breuil M. C. Bizouard F. Léonard J.. (2015). The diversity of the N_2_O reducers matters for the N_2_O:N_2_ denitrification end-product ratio across an annual and a perennial cropping system. Front. Microbiol. 6:971. doi: 10.3389/fmicb.2015.00971, PMID: 26441904 PMC4585238

[ref25] Edgar R. C. (2004). MUSCLE: multiple sequence alignment with high accuracy and high throughput. Nucleic Acids Res. 32, 1792–1797. doi: 10.1093/nar/gkh340, PMID: 15034147 PMC390337

[ref26] Francis C. A. Roberts K. J. Beman J. M. Santoro A. E. Oakley B. B. (2005). Ubiquity and diversity of ammonia-oxidizing archaea in water columns and sediments of the ocean. Proc. Natl. Acad. Sci. USA 102, 14683–14688. doi: 10.1073/pnas.0506625102, PMID: 16186488 PMC1253578

[ref27] Fu Q. Clark I. M. Zhu J. Hu H. Hirsch P. R. (2018). The short-term effects of nitrification inhibitors on the abundance and expression of ammonia and nitrite oxidizers in a long-term field experiment comparing land management. Biol. Fertil. Soils 54, 163–172. doi: 10.1007/s00374-017-1249-2

[ref28] Hartmann M. Frey B. Mayer J. Mäder P. Widmer F. (2015). Distinct soil microbial diversity under long-term organic and conventional farming. ISME J. 9, 1177–1194. doi: 10.1038/ismej.2014.210, PMID: 25350160 PMC4409162

[ref29] Hatzenpichler R. (2012). Diversity, physiology, and niche differentiation of ammonia-oxidizing archaea. Appl. Environ. Microbiol. 78, 7501–7510. doi: 10.1128/AEM.01960-12, PMID: 22923400 PMC3485721

[ref30] He S. Li Y. Mu H. Zhao Z. Wang J. Liu S.. (2021). Ammonium concentration determines differential growth of comammox and canonical ammonia-oxidizing prokaryotes in soil microcosms. Appl. Soil Ecol. 157:103776. doi: 10.1016/j.apsoil.2020.103776

[ref31] Hewitt A. E. (2010). New Zealand Soil classification. Lincoln, NZ: Manaaki Whenua Press.

[ref32] Hofstra J. (1977). Chlorate toxicity and nitrate reductase activity in tomato plants. Physiol. Plant 41, 65–69. doi: 10.1111/j.1399-3054.1977.tb01524.x

[ref33] Hornek R. Pommerening-Röser A. Koops H.-P. Farnleitner A. H. Kreuzinger N. Kirschner A.. (2006). Primers containing universal bases reduce multiple *amoA* gene specific DGGE band patterns when analysing the diversity of beta-ammonia oxidizers in the environment. J. Microbiol. Methods 66, 147–155. doi: 10.1016/j.mimet.2005.11.001, PMID: 16343671

[ref34] Hsu P. C. L. Di H. J. Cameron K. Podolyan A. Chau H. Luo J.. (2022). Comammox *Nitrospira* clade B is the most abundant complete ammonia oxidizer in a dairy pasture soil and inhibited by dicyandiamide and high ammonium concentrations. Front. Microbiol. 13:1048735. doi: 10.3389/fmicb.2022.1048735, PMID: 36578577 PMC9791190

[ref35] Huelsenbeck J. P. Ronquist F. (2001). MRBAYES: Bayesian inference of phylogenetic trees. Bioinformatics 17, 754–755. doi: 10.1093/bioinformatics/17.8.754 11524383

[ref36] Hynes R. K. Knowles R. (1983). Inhibition of chemoautotrophic nitrification by sodium chlorate and sodium chlorite: a reexamination. Appl. Environ. Microbiol. 45, 1178–1182. doi: 10.1128/aem.45.4.1178-1182.1983, PMID: 16346262 PMC242435

[ref37] Juliette L. Y. Hyman M. R. Arp D. J. (1993). Mechanism-based inactivation of ammonia monooxygenase in *Nitrosomonas europaea* by allylsulfide. Appl. Environ. Microbiol. 59, 3728–3735. doi: 10.1128/aem.59.11.3728-3735.1993, PMID: 16349087 PMC182524

[ref38] Kits K. D. Jung M. Y. Vierheilig J. Pjevac P. Sedlacek C. J. Liu S. R.. (2019). Low yield and abiotic origin of N_2_O formed by the complete nitrifier *Nitrospira inopinata*. Nat. Commun. 10:1836. doi: 10.1038/s41467-019-09790-x, PMID: 31015413 PMC6478695

[ref39] Kits K. D. Sedlacek C. J. Lebedeva E. V. Han P. Bulaev A. Pjevac P.. (2017). Kinetic analysis of a complete nitrifier reveals an oligotrophic lifestyle. Nature 549, 269–272. doi: 10.1038/nature23679, PMID: 28847001 PMC5600814

[ref40] Koch H. van Kessel M. A. H. J. Lücker S. (2019). Complete nitrification: insights into the ecophysiology of comammox *Nitrospira*. Appl. Microbiol. Biotechnol. 103, 177–189. doi: 10.1007/s00253-018-9486-3, PMID: 30415428 PMC6311188

[ref41] Könneke M. Bernhard A. E. de La Torre J. R. Walker C. B. Waterbury J. B. Stahl D. A. (2005). Isolation of an autotrophic ammonia-oxidizing marine archaeon. Nature 437, 543–546. doi: 10.1038/nature03911, PMID: 16177789

[ref42] Li C. Y. Hu H. W. Chen Q. L. Chen D. L. He J. Z. (2019). Comammox *Nitrospira* play an active role in nitrification of agricultural soils amended with nitrogen fertilizers [article]. Soil Biol. Biochem. 138:107609. doi: 10.1016/j.soilbio.2019.107609

[ref43] Li C. Hu H. W. Chen Q. L. Chen D. He J.-Z. (2020). Growth of comammox *Nitrospira* is inhibited by nitrification inhibitors in agricultural soils. J. Soils Sediments 20, 621–628. doi: 10.1007/s11368-019-02442-z

[ref44] Li C. Hu H. W. Chen Q. L. Yan Z. Z. Nguyen B. A. T. Chen D.. (2021). Niche specialization of comammox *Nitrospira* clade a in terrestrial ecosystems. Soil Biol. Biochem. 156:108231. doi: 10.1016/j.soilbio.2021.108231

[ref45] Nicol G. W. Leininger S. Schleper C. Prosser J. I. (2008). The influence of soil pH on the diversity, abundance and transcriptional activity of ammonia oxidizing archaea and bacteria. Environ. Microbiol. 10, 2966–2978. doi: 10.1111/j.1462-2920.2008.01701.x, PMID: 18707610

[ref46] Oksanen J. Blanchet F. G. Friendly M. Kindt R. Legendre P. McGlinn D.. (2022). *Vegan: community ecology package* (version 2.6-4) Available at: https://github.com/vegandevs/vegan

[ref47] Ouyang Y. Norton J. M. Stark J. M. Reeve J. R. Habteselassie M. Y. (2016). Ammonia-oxidizing bacteria are more responsive than archaea to nitrogen source in an agricultural soil. Soil Biol. Biochem. 96, 4–15. doi: 10.1016/j.soilbio.2016.01.012

[ref48] Palomo A. Dechesne A. Pedersen A. G. Smets B. F. (2022). Genomic profiling of *Nitrospira* species reveals ecological success of comammox *Nitrospira*. Microbiome 10:204. doi: 10.1186/s40168-022-01411-y, PMID: 36451244 PMC9714041

[ref49] Palomo A. Pedersen A. G. Fowler S. J. Dechesne A. Sicheritz-Pontén T. Smets B. F. (2018). Comparative genomics sheds light on niche differentiation and the evolutionary history of comammox Nitrospira. ISME J. 12, 1779–1793. doi: 10.1038/s41396-018-0083-3, PMID: 29515170 PMC6018701

[ref50] Park S. Bae W. (2009). Modeling kinetics of ammonium oxidation and nitrite oxidation under simultaneous inhibition by free ammonia and free nitrous acid. Process Biochem. 44, 631–640. doi: 10.1016/j.procbio.2009.02.002

[ref51] Pester M. Maixner F. Berry D. Rattei T. Koch H. Lücker S.. (2014). *NxrB* encoding the beta subunit of nitrite oxidoreductase as functional and phylogenetic marker for nitrite-oxidizing *Nitrospira*. Environ. Microbiol. 16, 3055–3071. doi: 10.1111/1462-2920.12300, PMID: 24118804

[ref52] R Core Team (2022). R: a language and environment for statistical computing. Vienna: R Foundation for Statistical Computing.

[ref53] Ravishankara A. Daniel J. S. Portmann R. W. (2009). Nitrous oxide (N_2_O): the dominant ozone-depleting substance emitted in the 21st century. Science 326, 123–125. doi: 10.1126/science.1176985, PMID: 19713491

[ref54] Rungvetvuthivitaya M. Song R. Campbell M. Ray C. (2014). A kinetic study of nitrification inhibition in water distribution systems using low levels of chlorite. J. Water Supply Res. Technol. AQUA 63, 497–506. doi: 10.2166/aqua.2014.135

[ref55] Sakoula D. Koch H. Frank J. Jetten M. S. van Kessel M. A. Lücker S. (2021). Enrichment and physiological characterization of a novel comammox *Nitrospira* indicates ammonium inhibition of complete nitrification. ISME J. 15, 1010–1024. doi: 10.1038/s41396-020-00827-4, PMID: 33188298 PMC8115096

[ref56] Shen T. Stieglmeier M. Dai J. Urich T. Schleper C. (2013). Responses of the terrestrial ammonia-oxidizing archaeon *ca. Nitrososphaera viennensis* and the ammonia-oxidizing bacterium *Nitrosospira multiformis* to nitrification inhibitors. FEMS Microbiol. Lett. 344, 121–129. doi: 10.1111/1574-6968.12164, PMID: 23617238

[ref57] Shi X. Hu H.-W. Wang J. He J.-Z. Zheng C. Wan X.. (2018). Niche separation of comammox *Nitrospira* and canonical ammonia oxidizers in an acidic subtropical forest soil under long-term nitrogen deposition. Soil Biol. Biochem. 126, 114–122. doi: 10.1016/j.soilbio.2018.09.004

[ref58] Soil Survey S. (2014). Keys to Soil taxonomy. Washington DC: United States Department of Agriculture (USDA) and Natural Resources Conservation Service.

[ref59] Subbarao G. V. Ito O. Sahrawat K. L. Berry W. L. Nakahara K. Ishikawa T.. (2006). Scope and strategies for regulation of nitrification in agricultural systems-challenges and opportunities. Crit. Rev. Plant Sci. 25, 303–335. doi: 10.1080/07352680600794232

[ref60] Sun D. Tang X. Li J. Liu M. Hou L. Yin G.. (2022). Chlorate as a comammox *Nitrospira* specific inhibitor reveals nitrification and N_2_O production activity in coastal wetland. Soil Biol. Biochem. 173:108782. doi: 10.1016/j.soilbio.2022.108782

[ref61] Sun P. Zhang S. Wu Q. Zhu P. Ruan Y. Wang Q. (2021). pH and ammonium concentration are dominant predictors of the abundance and community composition of comammox bacteria in long-term fertilized Mollisol. Appl. Soil Ecol. 168:104139. doi: 10.1016/j.apsoil.2021.104139

[ref62] Tatari K. Gülay A. Thamdrup B. Albrechtsen H.-J. Smets B. F. (2017). Challenges in using allylthiourea and chlorate as specific nitrification inhibitors. Chemosphere 182, 301–305. doi: 10.1016/j.chemosphere.2017.05.005 28505572

[ref63] van Kessel M. A. Speth D. R. Albertsen M. Nielsen P. H. Op den Camp H. J. Kartal B.. (2015). Complete nitrification by a single microorganism. Nature 528, 555–559. doi: 10.1038/nature16459, PMID: 26610025 PMC4878690

[ref64] Wang Z. H. Cao Y. Q. Zhu-Barker X. Nicol G. W. Wright A. L. Jia Z. J.. (2019). Comammox *Nitrospira* clade B contributes to nitrification in soil. Soil Biol. Biochem. 135, 392–395. doi: 10.1016/j.soilbio.2019.06.004

[ref65] Wang D.-Q. Zhou C.-H. Nie M. Gu J.-D. Quan Z.-X. (2021). Abundance and niche specificity of different types of complete ammonia oxidizers (comammox) in salt marshes covered by different plants. Sci. Total Environ. 768:144993. doi: 10.1016/j.scitotenv.2021.144993 33736320

[ref66] Whelan S. Goldman N. (2001). A general empirical model of protein evolution derived from multiple protein families using a maximum-likelihood approach. Mol. Biol. Evol. 18, 691–699. doi: 10.1093/oxfordjournals.molbev.a003851, PMID: 11319253

[ref67] Wickham H. (2016). ggplot2: Elegant graphics for data analysis. New York: In Springer-Verlag.

[ref68] Xu S. Y. Wang B. Z. Li Y. Jiang D. Q. Zhou Y. T. Ding A. Q.. (2020). Ubiquity, diversity, and activity of comammox *Nitrospira* in agricultural soils. Sci. Total Environ. 706:135684. doi: 10.1016/j.scitoteuv.2019.136684 31862588

[ref69] Xu G. Xu X. Yang F. Liu S. (2011). Selective inhibition of nitrite oxidation by chlorate dosing in aerobic granules. J. Hazard. Mater. 185, 249–254. doi: 10.1016/j.jhazmat.2010.09.025, PMID: 20926188

[ref70] Yang Y. Daims H. Liu Y. Herbold C. W. Pjevac P. Lin J.-G.. (2020). Activity and metabolic versatility of complete ammonia oxidizers in full-scale wastewater treatment systems. MBio 11, e03175–e03119. doi: 10.1128/mBio.03175-19 32184251 PMC7078480

[ref71] Zerulla W. Barth T. Dressel J. Erhardt K. Horchler von Locquenghien K. Pasda G.. (2001). 3,4-Dimethylpyrazole phosphate (DMPP) – a new nitrification inhibitor for agriculture and horticulture. Biol. Fertil. Soils 34, 79–84. doi: 10.1007/s003740100380

[ref72] Zhao Y. Hu J. Yang W. Wang J. Jia Z. Zheng P.. (2021). The long-term effects of using nitrite and urea on the enrichment of comammox bacteria. Sci. Total Environ. 755:142580. doi: 10.1016/j.scitotenv.2020.142580, PMID: 33059137

[ref73] Zhao Z. R. Huang G. H. He S. S. Zhou N. Wang M. Y. Dang C. Y.. (2019). Abundance and community composition of comammox bacteria in different ecosystems by a universal primer set. Sci. Total Environ. 691, 146–155. doi: 10.1016/j.scitotenv.2019.07.131, PMID: 31319252

