# Supplementary material for: Nitrification inhibitor chlorate and nitrogen substrates differentially affect comammox Nitrospira in a grassland soil
Source: Front Microbiol. 2024 May 14;15:1392090. doi: 10.3389/fmicb.2024.1392090 (PMC11130707; doi:10.3389/fmicb.2024.1392090)
Supplement: Supplementary file 2 [file Presentation_1.pdf]

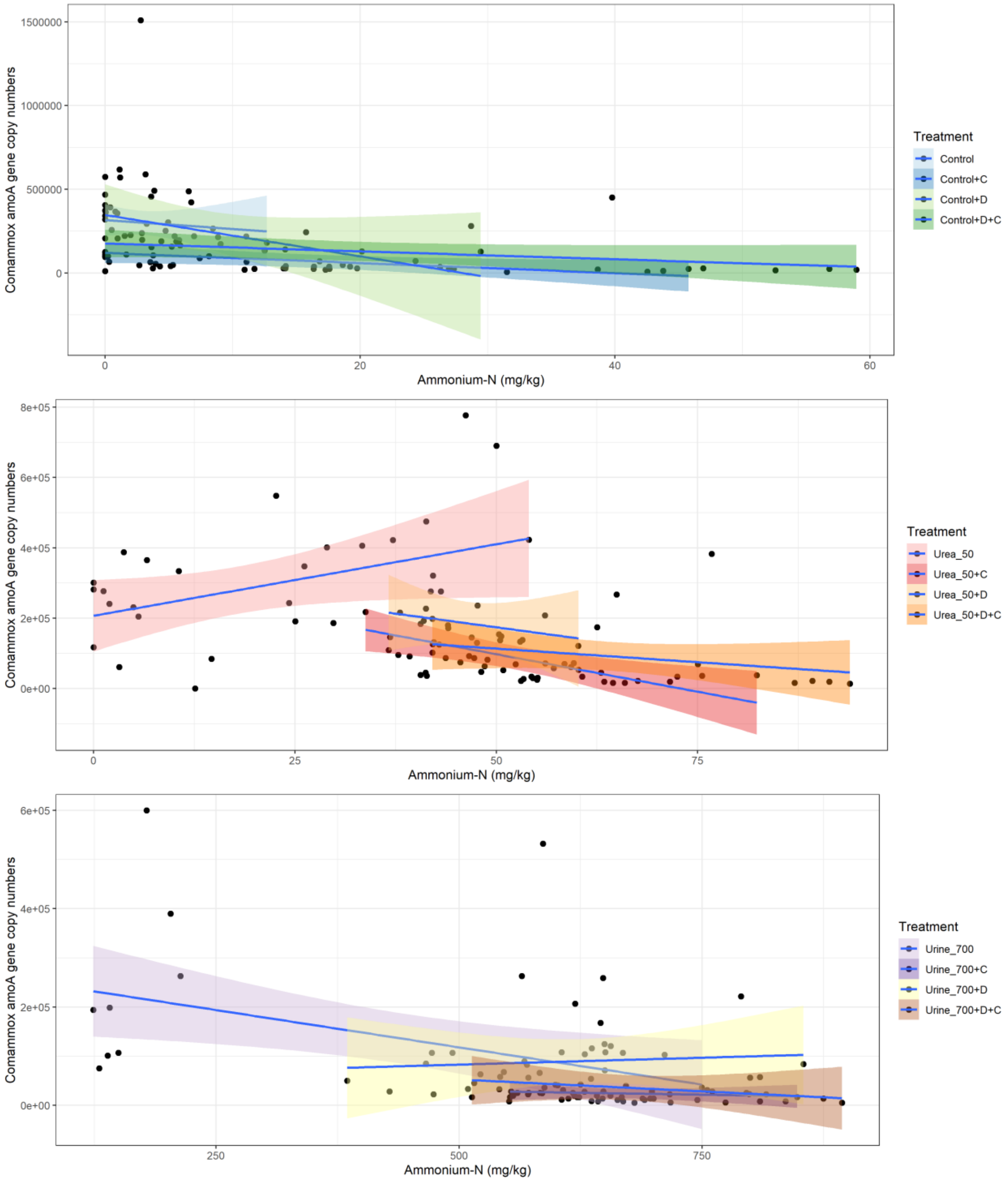

Figure S1: Relationship between comammox *Nitrospira amoA* gene abundance and ammonium levels across the 90-day experiment, split for the three nitrogen treatments. Shading around the lines represents the 95% confidence interval. DCD = D, Chlorate = C.

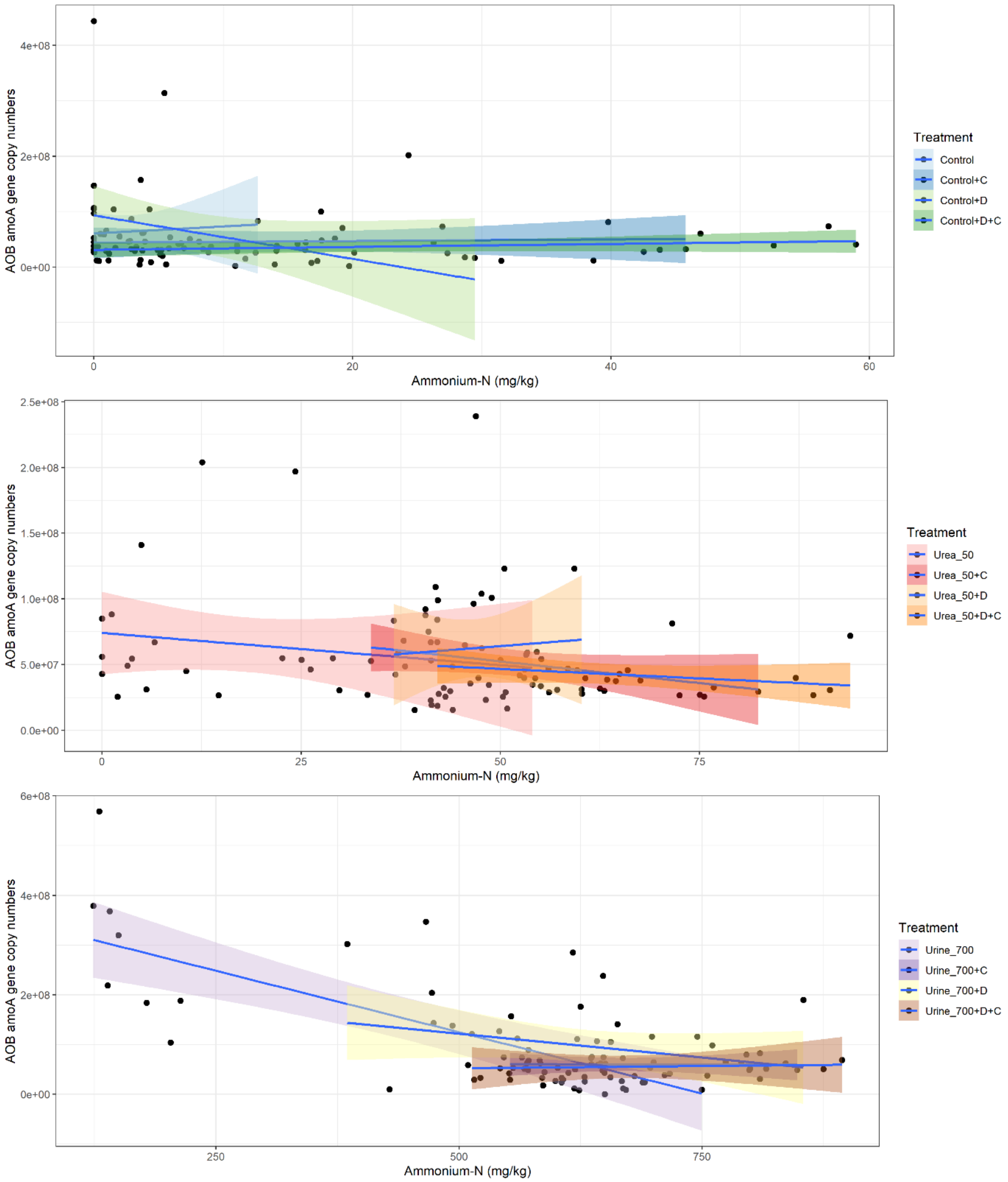

Figure S2: Relationship between AOB *amoA* gene abundance and ammonium levels across the 90-day experiment, split for the three nitrogen treatments. Shading around the lines represents the 95% confidence interval. DCD = D, Chlorate = C.

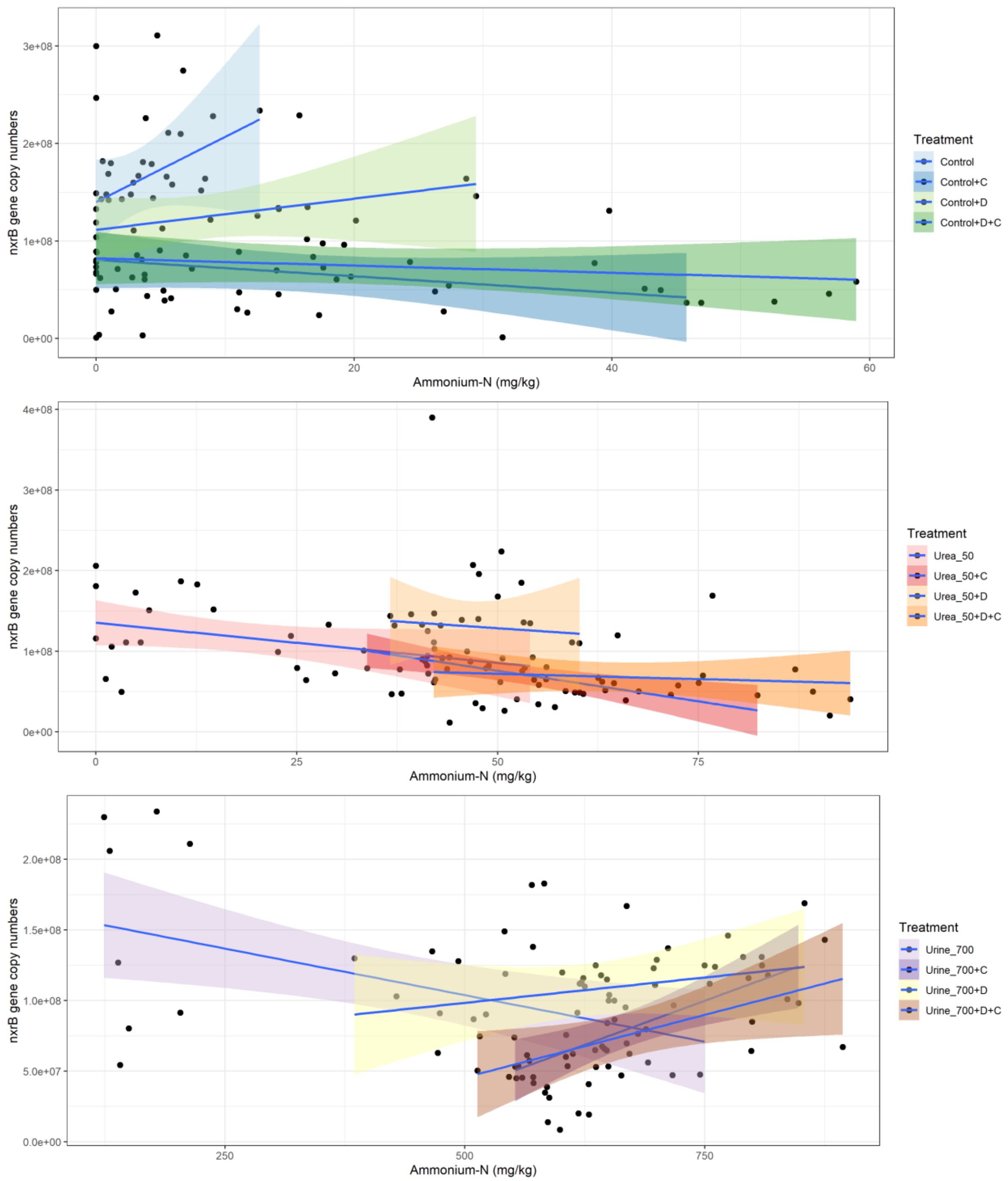

Figure S3: Relationship between *Nitrospira nxrB* gene abundance and ammonium levels across the 90-day experiment, split for the three nitrogen treatments. Shading around the lines represents the 95% confidence interval. DCD = D, Chlorate = C.

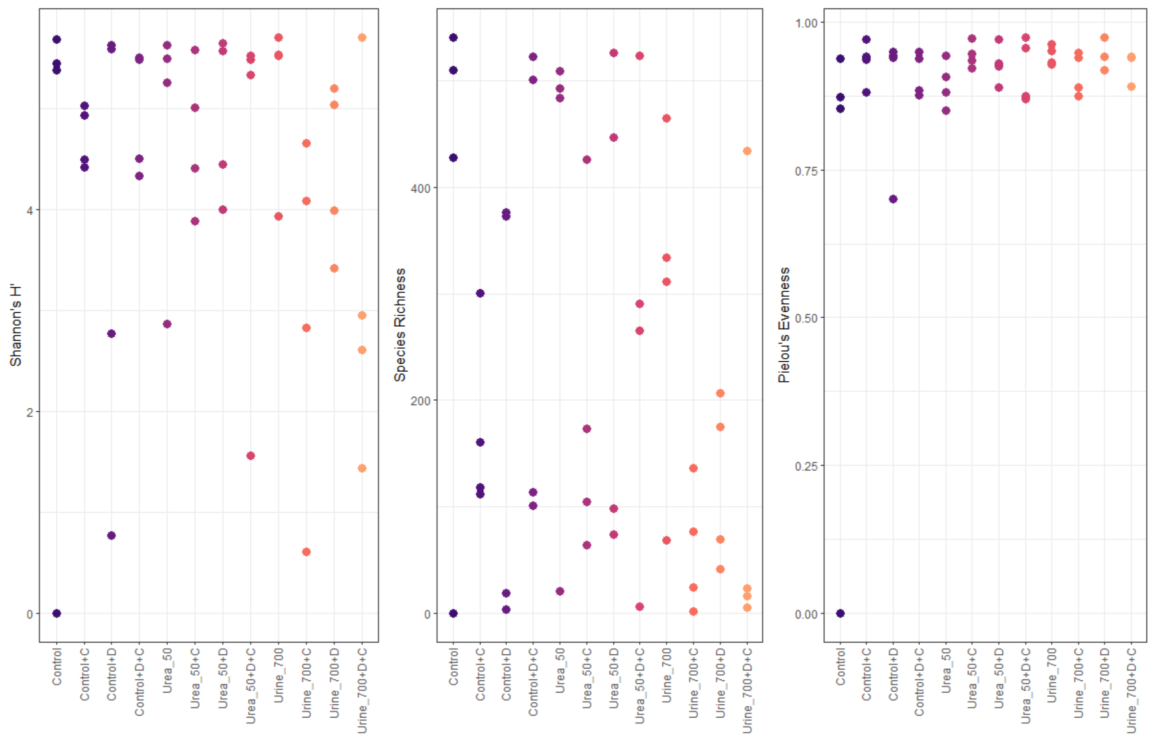

Figure S4: Alpha diversity plots for the comammox *Nitrospira amoA* gene across all treatments using Shannon's H`, species richness and Pielou's evenness indices. DCD = D, Chlorate = C.

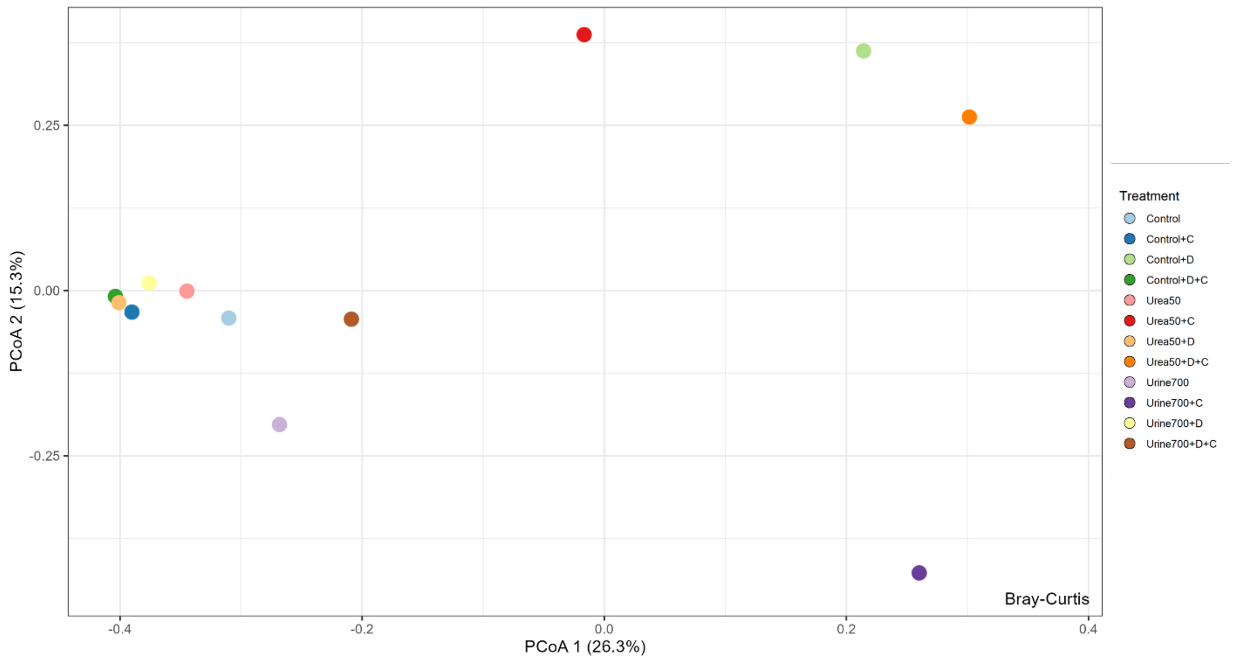

Figure S5: Principal coordinate analysis (PCoA) plot for comammox *Nitrospira amoA* gene across all treatments using the Bray-Curtis dissimilarity index (beta diversity). The variance explained by the PCoA axis is shown in parentheses. DCD = D, Chlorate = C.
